# Does modulation of the endocannabinoid system have potential therapeutic utility in cerebellar ataxia?

**DOI:** 10.1113/JP271106

**Published:** 2016-06-08

**Authors:** G. J. Stephens

**Affiliations:** ^1^ School of PharmacyUniversity of ReadingReadingRG6 6AJUK

## Abstract

Cerebellar ataxias represent a spectrum of disorders which are, however, linked by common symptoms of motor incoordination and typically associated with deficiency in Purkinje cell firing activity and, often, degeneration. Cerebellar ataxias currently lack a curative agent. The endocannabinoid (eCB) system includes eCB compounds and their associated metabolic enzymes, together with cannabinoid receptors, predominantly the cannabinoid CB_1_ receptor (CB_1_R) in the cerebellum; activation of this system in the cerebellar cortex is associated with deficits in motor coordination characteristic of ataxia, effects which can be prevented by CB_1_R antagonists. Of further interest are various findings that CB_1_R deficits may also induce a progressive ataxic phenotype. Together these studies suggest that motor coordination is reliant on maintaining the correct balance in eCB system signalling. Recent work also demonstrates deficient cannabinoid signalling in the mouse ‘ducky^2J^’ model of ataxia. In light of these points, the potential mechanisms whereby cannabinoids may modulate the eCB system to ameliorate dysfunction associated with cerebellar ataxias are considered.

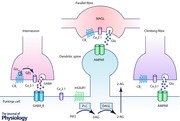

**Abbreviations**
2‐AG: 2‐arachidonylglycerol
CBD: cannabidiol
CBDV: cannabidivarin
CF: climbing fibre
eCB: endocannabinoid
GPCR: G protein‐coupled receptor
IN: interneuron
LTD: long term depression
MAGL: monoacylglycerol lipase
PC: Purkinje cell
pCB: phytocannabinoid
PF: parallel fibre
SCA: spinocerebellar ataxia
Δ^9^‐THC: tetrahydrocannabinol
VDCC: voltage‐dependent Ca^2+^ channel

## Cerebellar ataxias are a diverse group of disorders lacking a therapeutic agent

Cerebellar ataxias comprise a group of progressive neurological diseases associated with deficits in motor coordination and are typically associated with dysfunction and/or degeneration of Purkinje cells (PCs), the sole efferent output of the cerebellar cortex. There are a range of acquired ataxias and different hereditary forms of the disease (Klockgether, 2011). Thus, ataxia can be acquired from, amongst others, traumatic head injury, bacterial infection (meningitis or encephalitis), viral infection (chickenpox or measles), disruption of blood flow (stroke or transient ischaemic attack, haemorrhage), CNS disease (cerebral palsy or multiple sclerosis), sustained long‐term alcohol misuse, underactive thyroid gland and cancer autoimmune conditions (lupus), and can also be iatrogenic. Hereditary ataxias may be autosomal‐dominant diseases, including forms of spinocerebellar ataxia (SCA), several of which are associated with polyglutamine repeats in the dysfunctional protein; for example: ataxin 1 in SCA1; ataxin 2 in SCA2; Cacna1a encoding the voltage‐dependent Ca^2+^ channel (VDCC) Ca_V_2.1 subunit in SCA6 (also in episodic ataxia 2). There are also autosomal‐recessive diseases such as Friedreich's ataxia and ataxia telangiectasia associated with deficits in, respectively, the mitochondrial protein frataxin and a serine/threonine protein kinase termed ataxia telangiectasia mutated protein (Klockgether, 2011). Despite this range of causes and implicated proteins, deleterious effects are largely limited to the cerebellar cortex and are typically associated with cerebellar dysfunction and/or degeneration and are manifest as motor incoordination. This commonality of symptoms offers hope for providing treatment options; however, at present there is no known cure for cerebellar ataxia. There are treatments to ameliorate associated symptoms. For example, vitamin E and anti‐oxidants, such as co‐enzyme Q10 and its synthetic analogue idebenone, have been suggested to have some benefit, largely in Friedreich ataxia. However, as yet, such agents lack proven efficacy in controlled clinical trials (Cooper *et al*. 2008; Lynch *et al*. 2010), although some improvement in comparison to controls was seen in cross‐over trials, suggesting that patients with vitamin E‐deficient and co‐enzyme Q10‐deficient ataxia may receive some benefit (Cooper *et al*. 2008). In addition, administration of thyrotropin‐releasing hormone (TRH) was reported to ameliorate cerebellar ataxia in rolling Nagoya mice (Shibusawa *et al*. 2008) and the TRH analogue taltirelin is approved to improve motor performance in ataxic patients in Japan.

The elucidation of function of proteins associated with inherited ataxias within the cerebellar cortex may also lead to future therapeutic advances relevant across different forms of ataxia. Amongst target proteins, the Ca_V_2.1 (α1A) VDCC represents a widely studied protein. Ca_V_2.1 subunits are highly expressed in the cerebellum (Westenbroek *et al*. 1995; Kulik *et al*. 2004). In particular, Ca_V_2.1 is expressed postsynaptically in PCs (which led to the designation of these subunits as carriers of P‐type Ca^2+^ current (Mintz *et al*. 1992)), at presynaptic terminals of inhibitory interneurons (INs) arising from basket and stellate cells, and of excitatory, parallel fibres (PFs) and climbing fibres (CFs); such inputs regulate PC, and thus cerebellar cortex, output activity (Regehr & Mintz, 1994; Mintz *et al*. 1995; Stephens *et al*. 2001; Lonchamp *et al*. 2009). Several mouse Ca_V_2.1 mutants display ataxia (Pietrobon, 2010; Rajakulendran *et al*. 2012). Correspondingly, genetic deletion of Ca_V_2.1 is associated with a clear ataxic behavioural phenotype (Jun *et al*. 1999). Moreover, conditional PC‐specific Ca_V_2.1 knock‐down was shown to be sufficient to induce impaired synaptic transmission and ataxia (Mark *et al*. 2011; Todorov *et al*. 2012), the former study termed their mice ‘purky’. Cell‐specific work was extended to excitatory inputs into PCs, where it was shown that selective Ca_V_2.1 knockdown in PFs (arising from mossy fibre inputs) in so‐called ‘quirky’ mice, also gave rise to an ataxic phenotype (Maejima *et al*. 2013). Of further interest here, is that mutations which *increase* Ca_V_2.1 current also give impaired synaptic transmission and irregular PC firing (a cerebellar epitome predicted to lead to motor incoordination) (Gao *et al*. 2012); these reports suggest that correct VDCC activity must be maintained for PC firing fidelity. To add to purky and quirky, we also have ‘ducky’ mice (Barclay *et al*. 2001). Ducky, and the related ducky^2J^ (*du^2J^*) strain, have mutations predicted to lead to deficits in α2δ‐2 auxiliary VDCC subunit protein, which is expressed at high levels in normal cerebella (Cole *et al*. 2005) and is associated predominantly with Ca_V_2.1 in the cerebellum (Barclay *et al*. 2001). In different ducky strains, the ataxic phenotype is associated with a reduction in postsynaptic PC whole‐cell Ca^2+^ current (Brodbeck *et al*. 2002; Donato *et al*. 2006), together with irregular PC firing (Donato *et al*. 2006; Walter *et al*. 2006; Wang *et al*. 2013). Thus, several potential therapeutic targets have been suggested, largely confined to protein associated with inherited ataxias; however, as discussed above, as yet we have no cure for ataxia. The remainder of this review will focus on the potential to target the endocannabinoid (eCB) system to ameliorate cerebellar ataxia and, in particular, eCB compounds and their associated metabolic enzymes and G protein‐coupled CB_1_R, one of the most ubiquitously expressed proteins in the mammalian cerebellum, and a protein which also modulates Ca_V_2.1 activity at the presynapse.

## Cannabinoid signalling and its potential links to cerebellar ataxia

Cannabinoids represent a diverse number of compounds, including (i) eCBs, for example, the lipid mediator 2‐arachidonylglycerol (2‐AG), which is the major eCB in the cerebellum (Szabo *et al*. 2006); (ii) plant‐derived phytocannabinoids (pCBs), for example, the major herbal *Cannabis* constituents tetrahydrocannabinol (Δ^9^‐THC) and cannabidiol (CBD) (Hill *et al*. 2012 *a*); and (iii) exogenous synthetic agents, namely CB_1_R agonists, for example, WIN 55,212‐2, an aminoalkylindole derivative, and CP 55940, which is structurally related to tetrahydrocannabinol, and CB_1_R antagonists/inverse agonists, for example, rimonabant (Pertwee *et al*. 2010), and newer allosteric modulators, for example, Org27569 (Price *et al*. 2005) and PSNCBAM‐1 (Horswill *et al*. 2007).

**Figure 1:**
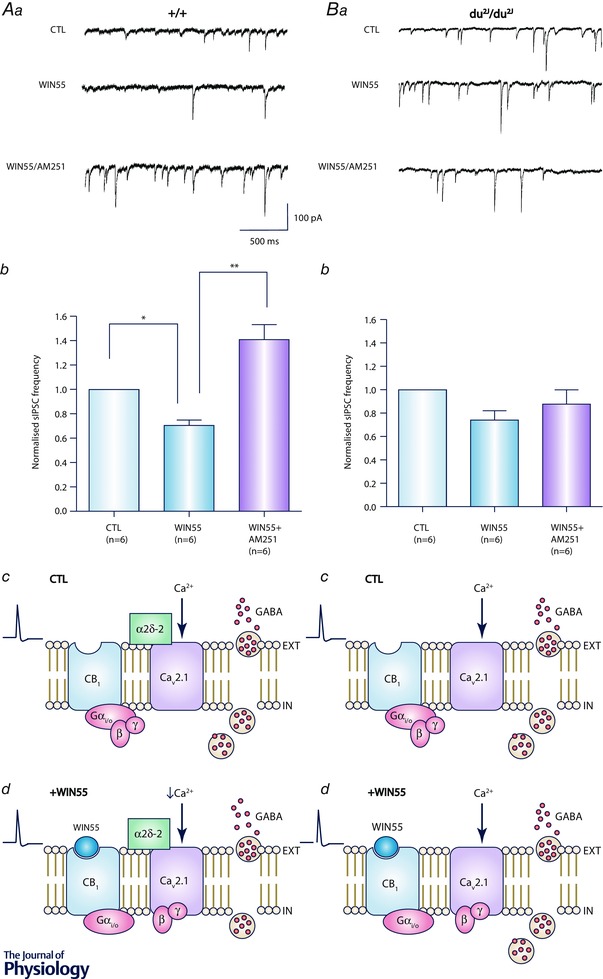
**Presynaptic CB_1_R modulation of inhibitory transmission at IN–PC synapses is deficient in ataxic ducky^2J^ mice** *Aa* and *Ba*, representative spontaneous inhibitory postsynaptic current (sIPSC) traces from +/+ (*Aa*) and *du^2J^/du^2J^* (*Ba*) PCs showing effect of WIN55 (5 μm), and also subsequent application of AM251 (2 μm). *Ab* and *Bb*, summary bar graphs showing that WIN55 significantly reduced, and AM251 significantly increased, normalized sIPSC frequency in +/+ (*Ab*), but was without effect in *du^2J^/du^2J^* (*Bb*), conditions. ^*^ *P* < 0.05; ^**^ *P* < 0.01; repeated measurement one‐way ANOVA followed by Tukey's honest significant difference test. *Ac* and *Bc*, summary diagrams for +/+ (*Ac*) and *du^2J^/du^2J^* (*Bc*) conditions. *Ad*, in wild‐type conditions, CB_1_R activation (i.e. +WIN55) causes the release of Gβγ subunit from CB_1_R and subsequent inhibitory coupling of Gβγ to Cav2.1 at the presynapse to inhibit the action potential‐evoked GABA release seen in control (CTL). *Bd*, by contrast, in *du^2J^/du^2J^* conditions, CB_1_R activation (i.e. +WIN55) has no effect on the GABA release seen in control (CTL). AM251 effects were also absent (see Wang *et al*. 2013). Thus, we propose that at synapses lacking α2δ‐2 subunits (which associate predominantly with Ca_V_2.1 in the cerebellum; Barclay *et al*. 2001), normal CB_1_R modulation of Ca_V_2.1 is lacking. This deficit may relate to incorrect control of synaptic release by α2δ subunits (Hoppa *et al*. 2012); alternatively, it is possibly that lack of α2δ subunits may cause changes to CB_1_R‐mediated G protein inhibition of Ca_V_2.1.

Within the CNS, cannabinoids predominantly activate CB_1_Rs, which represent the most widespread G protein‐coupled receptor (GPCR) in the mammalian cerebellum (Herkenham *et al*. 1991; Tsou *et al*. 1997). CB_1_R expression is reported to be very low at PC cell bodies; rather, expression is high at excitatory PF inputs into PCs, reportedly with a perisynaptic over extrasynaptic and synaptic localization, with lower CB_1_R expression at CF inputs onto PC dendritic shafts (Kawamura *et al*. 2006; see Abstract Figure). CB_1_Rs are expressed at higher levels on presynaptic terminals of inhibitory INs, predominantly basket cells, but also stellate cells, which form a specialized region surrounding the PC axon initial segment known as the pinceau (the French word for paintbrush) (Tsou *et al*. 1997; Kawamura *et al*. 2006; Rodríguez‐Cueto *et al*. 2014). Presynaptic CB_1_Rs are activated by retrograde ‘on demand’ release of 2‐AG from postsynaptic PCs. The major effect of presynaptic CB_1_R activation is a suppression of neurotransmitter release, whereby activation of presynaptic CB_1_Rs inhibits action potential‐evoked and spontaneous inhibitory postsynaptic currents (IPSCs) at IN–PC synapses (see Fig. 1) or excitatory postsynaptic currents (EPSCs) at PF–PC and CF–PC synapses (Takahashi & Linden, 2000; Szabo *et al*. 2004; Kano *et al*. 2009). We have also used multi‐electrode array recording to demonstrate that CB_1_R ligand‐induced changes to cerebellar cortex network activity are mediated, at least in part, via effects on inhibitory synaptic transmission (Ma *et al*. 2008). CB_1_R activation has been widely associated with a number of different forms of short‐ and long‐term synaptic plasticities which modulate cerebellar learning (Kano *et al*. 2009; Ohno‐Shosaku & Kano, 2014). Thus, 2‐AG release mediates the short‐term suppression of inhibitory GABA release from IN terminals (depolarization‐induced suppression of inhibition) or excitatory glutamate release (depolarization‐induced suppression of excitation) (Szabo *et al*. 2006; Tanimura *et al*. 2009). Seminal work by Ito (1989) linked long term depression (LTD) by associative stimulation of PF and CF inputs to PCs, to motor learning in the cerebellum. It is also known that the metabotropic glutamate receptor 1 (mGluR1) pathway is critically involved in cerebellar development and LTD (Aiba *et al*. 1994), and it was further proposed that CF inputs into individual PCs were required for normal motor coordination (Chen *et al*. 1995). More recent work has established that cerebellar LTD is a postsynaptic phenomenon requiring 2‐AG release from PCs and activation of presynaptic CB_1_Rs at PF–PC synapses (Safo & Regehr, 2005). Here, mGluR1 activation drives 2‐AG release (Kano *et al*. 2009; see Abstract Figure). Thus, CB_1_Rs have a privileged position in the function and control of overall output of the cerebellar cortex and, as such, represent good potential targets to modulate dysfunctional signalling associated with cerebellar ataxias.

In cell lines and native neurons, CB_1_R activation causes pertussis toxin‐sensitive inhibition of Ca_V_2 family VDCCs, and can also activate inwardly rectifier K^+^ channels (Mackie & Hille, 1992; Twitchell *et al*. 1997; Guo & Ikeda, 2004). It is likely that CB_1_Rs couple to presynaptic Ca_V_2.1 (P/Q‐type) VDCCs at IN–PC synapses to reduce action potential evoked GABA release (Forti *et al*. 2000; Stephens *et al*. 2001; Lonchamp *et al*. 2009) and to Ca_V_2.1 (and to a lesser extent Ca_V_2.2 and Ca_V_2.3) at PF–PC synapses to reduce action potential‐evoked glutamate release (Brown *et al*. 2004); these effects are most likely mediated by direct binding of G protein Gβγ subunits to VDCCs (Abstract Figure and Fig. 1).

CB_1_R agonists also cause clear reductions in frequency of ‘miniature’ IPSCs at IN–PC synapses (Takahashi & Linden, 2000; Yamasaki *et al*. 2006; Ma *et al*. 2008), consistent with an inhibition of action potential‐independent GABA release. These effects are proposed to occur downstream of actions on voltage‐dependent ion channels and are also consistent with direct effects on the synaptic release machinery, and also may be mediated by Gβγ subunits (Stephens, 2009). By contrast, CB_1_R agonist effects on miniature EPSCs at PF–PC synapses were only apparent when extracellular Ca^2+^ levels were increased (Yamasaki *et al*. 2006). Moreover, CB_1_R antagonists/inverse agonists such as AM251, rimonabant and the pCB Δ^9^‐tetrahydrocannabivarin all increase inhibitory GABA release at IN–PC synapses (Ma *et al*. 2008). Such effects are consistent with the presence of a strong, modulatable eCB tone in the cerebellum (Kreitzer *et al*. 2002; Galante & Diana, 2004), which provides further opportunity for therapeutic intervention in cerebellar ataxia.

Importantly, activation of presynaptic CB_1_Rs by synthetic cannabinoids and eCBs has been shown to promote cerebellar dysfunction, causing severe motor incoordination and modelling cerebellar ataxia (Lichtman *et al*. 1998; DeSanty & Dar, 2001; Patel & Hillard, 2001); in these studies pre‐treatment with a CB_1_R antagonist or CB_1_R antisense prevented the induction of an ataxic phenotype. Such data suggest that CB_1_R antagonism may be useful in the pathogenic situation. In comparison to administration of CB_1_R ligands, data with CB_1_R knock‐out mice are somewhat more equivocal. Thus, young/mature CB_1_R deficient mice are reported not to exhibit clear motor discoordination or changes to gait (Steiner *et al*. 1999; Kishimoto & Kano, 2006); however, deficits in motor function were reported in mature and older mice, in comparison to unaffected younger mice, in rotarod tests (Bilkei‐Gorzo *et al*. 2005). One interpretation of these studies is that a progressive ataxic pathogenesis may be associated with long‐term loss of CB_1_R. One common feature of CB_1_R knock‐out mice, chronic marijuana users or animals administered CB_1_R agonists is a reported deficit in delay eyeblink conditioning, a cerebellar‐dependent, motor learning process (Kishimoto & Kano, 2006; Skosnik *et al*. 2008; Steinmetz & Freeman, 2010). These data are consistent with CB_1_R controlling discrete motor function. Kishimoto & Kano (2006) also report that pharmacological block of CB_1_R had no effect on motor function in wild‐type mice; however, it is also important to point out that CB_1_R deficiency and/or lack of effect of CB_1_R antagonism in a non‐pathogenic situation does not preclude a role in disease; for example, whilst SR141617A (rimonabant) reversed CB_1_R‐induced dysfunction, it had no effects itself on motor incoordination in non‐ataxic animals (Lichtman *et al*. 1998; DeSanty & Dar, 2001). Together, these data suggest that CB_1_Rs modulate cerebellar circuitry in ataxic disease, potentially with a progressive onset of effect. Therefore, targeting CB_1_Rs may be beneficial in modulating motor incoordination in cerebellar ataxia, as discussed more fully below.

## An ataxic mouse model has deficient CB_1_R signalling

Whilst the role of ion channels (in particular, Ca_V_2.1) has been broadly studied in animal models of ataxia, there has been much less work on the presynaptic receptors that modulate neurotransmitter release and the postsynaptic receptors responsible for onward signalling in such models. A study in Ca_V_2.1 mutant tottering mice by Zhou and co‐workers reported that presynaptic inhibition mediated by GABA_B_ or α2‐adrenoceptor GPCRs was enhanced at excitatory PF–PC synapses, although this may be as consequence of a switch to a reliance on Ca_V_2.2 (N‐type) channels for transmitter release (Zhou *et al*. 2003). Tottering mice also had a reduction in GABA_A_ receptor expression, with specific deficits in granule cells (Kaja *et al*. 2007). We have shown that ataxic *du^2J^* mutant mice exhibit increased irregularity of PC and, to a lesser extent, granule cell firing in multi‐electrode array recordings from cerebellar brain slices (Wang *et al*. 2013). Of note, clear effects on PC firing regularity in *du^2J^*/*du^2J^* mice were not seen in heterozygous +/*du^2J^* mice, and the latter also lacked a clear behavioural ataxic phenotype. Importantly, the CB_1_R‐mediated inhibition at IN–PC synapses seen in litter‐matched controls was completely absent in both +/*du^2J^* and *du^2J^*/*du^2J^* mice. These data demonstrate that ataxic α2δ‐2‐deficient mice have aberrant presynaptic CB_1_R‐mediated signalling. The question arises as to whether deficiency in CB_1_R‐mediated signalling is involved in ataxia pathogenesis or whether it occurs as a result of the disease. It appears that, in this model, both alleles need to be affected in order for an ataxic phenotype to be seen (Wang *et al*. 2013), and thus progressive deficits may be associated with *du^2J^* mice. We saw no changes in PC firing regularity in response to CB_1_R ligands in wild‐type or *du^2J^* mice, consistent with a lack of postsynaptic CB_1_R effects in this model. We propose that such deficits occur due to compromised Ca^2+^ channel activity consequent to reduced presynaptic α2δ‐2 expression in *du^2J^* mice (Fig. 1). In this regard, α2δ‐2 subunits have been shown to be essential not only for Ca^2+^ channel trafficking (Dolphin, 2012), but also for synaptic function, the latter by increasing transmitter release probability and also protecting release from inhibitory effects of intracellular Ca^2+^ chelators (Hoppa *et al*. 2012).

There are few studies measuring CB_1_R expression in ataxic animals; we reported no clear changes in expression in the cerebellar cortex of +/*du^2J^* and *du^2J^*/*du^2J^* mice (Wang *et al*. 2013). In a recent study, post‐mortem cerebellar tissue from patients with SCAs, CB_1_R (and CB_2_R) expression was generally up‐regulated in PCs, and also in glial cells (Rodríguez‐Cueto *et al*. 2014 *a*). Of interest here was that CB_1_R expression was reported in PC soma and pinceau in SCA patients, but was confined largely to the pinceau in control patients. It is possible that upregulated postsynaptic CB_1_R expression may affect 2‐AG release in SCA patients; however, Rodriguez‐Cueto and co‐workers suggest that this CB_1_R expression is associated with degenerating PCs and may represent a marker for degeneration and/or a protective response against such degeneration. A further study in SCA patients reported an up‐regulation of eCB degradative fatty acid amide hydrolase and monoacylglycerol lipase (MAGL) enzymes (Rodríguez‐Cueto *et al*. 2014 *b*), proposed to lead to reduced eCB levels in disease. In these studies, a compensatory up‐regulation of cannabinoid receptor expression may occur as a consequence of reduced eCB levels; alternatively, it may be argued that eCBs are suppressed in order not to overactivate the system. Thus, it may also be possible to target eCB metabolizing enzymes for future therapeutic development. In this regard, potential avenues to increase 2‐AG include inhibition of MAGL, localized predominantly to the PF terminal (Tanimura *et al*. 2012), or activation of the biosynthetic enzyme diacylglycerol lipase‐α (DAGLα) localized predominantly to the base of postsynaptic dendritic spines (Yoshida *et al*. 2006) (see Abstract Figure). It is also clear that we will need to investigate potential changes to eCB signalling in different animal models of ataxia to inform development of the most useful therapeutic strategies and also to determine if any such changes represent useful markers for different forms of ataxia.

## Do cannabinoids have therapeutic utility in cerebellar ataxia?

There are anecdotal reports that cannabis smokers can achieve symptom relief for several CNS disorders. Such evidence has fuelled the investigation of use of CB_1_R agonists as potential neuroprotective agents for a range of conditions including epilepsy, neurodegenerative diseases such as Alzheimer's, Parkinson's and Huntington's disease and appetitive disorders (Fernández‐Ruiz *et al*. 2011; Hill *et al*. 2012 *a*). Earlier evidence for ataxia is largely confined to two case studies which suggest that oral Δ^9^‐THC or marijuana improved motor coordination in some multiple sclerosis patients (Clifford, 1983; Meinck *et al*. 1989). At the clinical level, synthetic Δ^9^‐THC has been used in management of nausea, emesis and pain, and nabiximols (Sativex) (containing ∼1:1 Δ^9^‐THC:CBD) represents the first phytocannabinoid medicine, used as an oromucosal spray for pain and spasticity associated with multiple sclerosis (Hill *et al*. 2012 *a*); Sativex was also stated to delay onset of ataxia symptoms in the Medicines and Healthcare products Regulatory Agency (MHRA) Public Information Report UK/H/961/01/DC. Such reports contributed to fuelling a major review on the clinical effects of cannabinoids in ataxia associated with multiple sclerosis (Mills *et al*. 2007); although cannabinoids showed promise, it was concluded that better standardized measures of ataxia were needed to fully establish the utility of cannabis‐based medicines in ataxia. The role of cannabinoids in disease‐associated movement disorders and tremor has been further discussed more recently by Arjmand *et al*. (2015) and Kluger *et al*. (2015), who similarly concluded that further work on cannabinoids in different models of ataxia is warranted. In this regard, an interesting recent report suggests that a ‘Sativex‐like’ combination of Δ^9^‐THC and CBD, as well as the individual administration of Δ^9^‐THC or CBD, was able to improve motor deficits in a viral model of multiple sclerosis (Feliú *et al*. 2015). Clearly, studies which suggest CB_1_R activation may be useful in cerebellar ataxia contrast to preclinical data where CB_1_R agonists induce an ataxic phenotype (Lichtman *et al*. 1998; DeSanty & Dar, 2001; Patel & Hillard, 2001); however, these data do support the hypothesis that maintaining the correct balance in eCB system signalling is a major factor for proper control of motor coordination. This hypothesis is further supported by data from Ca_V_2.1 mutants described above, where both decreases (Maejima *et al*. 2013) and increases in Ca^2+^ current (Gao *et al*. 2012) can produce an ataxia phenotype; moreover, deficits in delay eyeblink conditioning are reported for both CB_1_R agonists and CB_1_R antagonists/inverse agonists using the same experimental design (Steinmetz & Freeman, 2010). CB_1_R agonists have also been shown to possess functional selectively or ‘biased agonism’, whereby different ligands (including eCBs) preferentially activate different CB_1_R signalling pathways (Laprairie *et al*. 2014; Khajehali *et al*. 2015). Whilst, as argued above, it is likely that CB_1_Rs act predominantly on presynaptic Ca_V_2.1 to reduce transmitter release in the cerebellar cortex, alternative signalling pathways include inhibition of cAMP or stimulation of phosphorylation of signal regulated kinases (Howlett *et al*. 2010). Thus, it could be argued that by using knowledge of biased agonism that we can target specific pathways associated with diseases, including cerebellar ataxia. Finally here, CB_1_R agonists such as Δ^9^‐THC have also been proposed to possess anti‐oxidant (Hampson *et al*. 1998) and anti‐inflammatory (although largely CB_2_R‐mediated) (Fernández‐Ruiz *et al*. 2011) properties; in common with other degenerative diseases, such properties may benefit the amelioration of cerebellar ataxia symptoms.

The demonstration that pre‐treatment with a CB_1_R antagonist prevents the induction of motor incoordination by CB_1_R agonists (DeSanty & Dar, 2001; Patel & Hillard, 2001) suggests that CB_1_R antagonists/inverse agonists may be protective in ataxia. The archetypal agent rimonabant was introduced as an anti‐obesity agent, but was withdrawn due to fears of increased suicidality and depression in patients (Nathan *et al*. 2011). Since then, therapeutic development of CB_1_R antagonists/inverse agonists has largely been put on hold. Interesting potential alternatives are CB_1_R negative allosteric antagonists, such as Org‐27569 and PSNCBAM‐1. These compounds have a somewhat unique pharmacological profile as they increase orthosteric agonist binding, but decrease agonist activity; more intriguingly, allosteric antagonism action is ligand‐dependent and also shows biased antagonism for different signalling pathways (Baillie *et al*. 2013). We have shown that such functional selectivity for PSNCBAM‐1 extends to effects on orthosteric ligands at IN–PC synapses in the cerebellar cortex (Wang *et al*. 2011); thus, PSNCBAM‐1 attenuated CP55940 agonist and AM251 antagonist effects, but had no clear effects against WIN 55,212‐2. Moreover, when applied alone, PSNCBAM‐1 was not associated with potentially deleterious effects on eCB tone, a concern associated with use of CB_1_R antagonists/inverse agonists such as rimonabant. These studies indicate that exogenous allosteric CB_1_R ligands have potential to fine tune eCB orthosteric agonist effects in a ligand‐ and/or cell signalling‐selective manner within the cerebellar cortex; moreover, biased antagonism effects may allow for further useful therapeutic development.

This review has focused on cannabinoids as agents acting on the eCB system in the cerebellum; moreover, the modulation of CB_1_Rs has been highlighted. It may transpire that, for exogenous compounds, targeting alternative modes of action offers improved therapeutic potential in diseases such as ataxia. The last few years have seen increasing calls for the use of medical marijuana to treat a range of disorders; of course, use of marijuana is intimately associated with psychoactive effects of the CB_1_R partial agonist Δ^9^‐THC. Therapeutically, a more attractive option may be use of non‐psychoactive pCBs. Thus, CBD and cannabidivarin (CBDV) have reported utility in epilepsy and, potentially, other CNS disorders (Hill *et al*. 2012 *b*, 2013; Devinsky *et al*. 2014). The demonstration that Sativex can improve motor activity in multiple sclerosis (Feliú *et al*. 2015) is consistent with beneficial effects of a CB_1_R activator (Δ^9^‐THC) in combination with CBD as a potential ameliorating agent for unwanted Δ^9^‐THC effects (McPartland *et al*. 2015); for example, CBD alone was also effective in improving motor deficits, potentially via an action on peroxisome proliferator‐activated receptor γ (PPARγ) receptors (Feliú *et al*. 2015). In this regard, CBD has recently been awarded orphan drug status for the severe childhood epilepsy Dravet syndrome and is currently progressing well through clinical trials. CBD and CBDV have only low affinity at CB_1_Rs and CBD has been proposed, amongst other possibilities, to act at alternative GPCRs or at transient receptor potential ion channels or, possibly, to augment eCB tone via effects on metabolic enzymes (Hill *et al*. 2012 *a*; McPartland *et al*. 2015). A recent study has proposed that CBD, as well as having CB_1_R‐independent actions, may also act as a CB_1_R negative allosteric antagonist (Laprairie *et al*. 2015); therefore, CBD may share useful properties of this class of agents discussed above. It is also of interest that the hypophagic effects of the allosteric antagonist Org27569 have been suggested to occur independently of CB_1_Rs (Ding *et al*. 2014; Gamage *et al*. 2014). Thus, the use of cannabinoids with CB_1_R‐independent and/or allosteric actions should also be considered.

In the future, it will be of interest in particular to test agents such as Sativex, and perhaps CBD as an individual compound, in cerebellar ataxia. There are a number of general points to consider, including whether deficits in CB_1_R‐mediated signalling are hallmark characteristics of different forms of ataxia, how best to target such deficits and whether aberrant cannabinergic signalling represents a useful biomarker for early or asymptomatic cerebellar ataxia. The answer to such questions will go some way to determining if modulation of the eCB system has therapeutic utility in cerebellar ataxia.

## Additional information

### Competing interests

The author has no conflict of interest.

### Funding

Work in my group included here was supported by The Wellcome Trust and an Ataxia UK Postgraduate Fellowship awarded to Xiaowei Wang, who also received a University of Reading Postgraduate Research Studentship Award and was co‐supervised by Prof. Benjamin Whalley. I also acknowledge the support of GW Pharmaceuticals in supplying material and associated grant funding during some of this work.
